# Characteristics of patients presenting with myocardial infarction with non-obstructive coronary arteries (MINOCA) in Poland: data from the ORPKI national registry

**DOI:** 10.1007/s11239-018-1794-z

**Published:** 2018-12-18

**Authors:** Tomasz Rakowski, Giuseppe De Luca, Zbigniew Siudak, Krzysztof Plens, Artur Dziewierz, Paweł Kleczyński, Tomasz Tokarek, Michał Węgiel, Marcin Sadowski, Dariusz Dudek

**Affiliations:** 1 Institute of Cardiology, Jagiellonian University Medical College, Kopernika 17 Street, 31-501 Kraków, Poland; 2 2nd Department of Cardiology and Cardiovascular Interventions, University Hospital, Kraków, Poland; 3 Division of Cardiology, Azienda Ospedaliera-Universitaria “Maggiore della Carità”, Eastern Piedmont University, Novara, Italy; 4 Jan Kochanowski University, Kielce, Poland; 5 KCRI, Kraków, Poland

**Keywords:** Myocardial infarction, Registries, Non-obstructive coronary artery disease, Coronary angiography

## Abstract

Myocardial infarction (MI) with non-obstructive coronary arteries (MINOCA) is an important clinical problem especially in the era of extensive utilization of coronary angiography in MI patients. Its pathophysiology is poorly understood which makes diagnostics and treatment of MINOCA challenging in everyday clinical practice. The aim of the study was to assess characteristics of MINOCA patients in Poland based on data from the Polish National ORPKI Registry. In 2016, 49,893 patients with non-ST-segment elevation (NSTEMI) or ST-segment elevation (STEMI) myocardial infarction entered the ORPKI registry. MINOCA was defined as a non-obstructive coronary artery disease (CAD) and a lack of previous coronary revascularization. MINOCA was identified in 3924 (7.8%) patients and clinical presentation was more often NSTEMI than STEMI (MINOCA: 78 vs. 22%; obstructive CAD 51.1 vs. 48.9%; p < 0.0001). MINOCA patients were younger and more often females with significantly lower rates of diabetes, smoking, arterial hypertension, kidney disease, previous MI and previous stroke comparing to patients with obstructive CAD. Myocardial bridge was visualized in angiography more often in the MINOCA group (2.2 vs. 0.4%; p < 0.0001). Additional coronary assessment inducing fractional flow reserve, intravascular ultrasound, optical coherence tomography was marginally (< 1%) used in both groups. Periprocedural mortality was lower in MINOCA group (0.13% vs. 0.95%; p < 0.0001). MINOCA patients represent a significant proportion of MI patients in Poland. Due to multiple potential causes, MINOCA should be considered rather as a working diagnosis after coronary angiography and further efforts should be taken to define the cause of MI in each individual patient.

## Highlights

- Myocardial infarction with non-obstructive coronary arteries (MINOCA) is an important clinical problem in the era of extensive usage of coronary angiography.
- Many of MINOCA causes are treatable, so additional diagnostics beyond coronary angiography is important for final diagnosis and treatment selection.
- In large-scale national registry patients with MINOCA represent a significant proportion of MI patients in Poland (7.8% of total myocardial infarction patients).
- Due to multiple potential causes, MINOCA should be considered rather as a working diagnosis after coronary angiography and further efforts should be taken to define the cause of MI in each individual patient.

## Introduction

Myocardial infarction (MI) with non-obstructive coronary arteries (MINOCA) is an important and common clinical problem as according to current knowledge, almost every patient with the diagnosis of MI should be referred to coronary angiography. An invasive strategy is also a frequent approach in unclear cases, in which after ruling out the obstructive coronary artery disease (CAD) further diagnostics should be applied. The pathophysiology of MINOCA is multifactorial and poorly understood with several proposed mechanisms. This makes diagnostics and treatment of MINOCA challenging in daily clinical practice. That is why the problem of MINOCA diagnostics was highlighted in the recent European Society of Cardiology Clinical Practice Guidelines on acute myocardial infarction in patients presenting with ST-segment elevation [1]. However, the problem of MINOCA was not explored in patients with MI in Poland. The purpose of the present analysis was to assess characteristics of MINOCA patients in Poland based on data from the Polish National Percutaneous Coronary Interventions Registry (ORPKI).

## Methods

Data on patients with the diagnosis of ST-segment elevation (STEMI) or non-ST-segment elevation myocardial infarction (NSTEMI) included into the ORPKI between January and December 2016 (12 months) were analyzed. The ORPKI is a national electronic database operated by the Jagiellonian University Medical College in Krakow collecting data on all percutaneous procedures in interventional cardiology performed in Poland [2, 3]. All medical procedures were performed according to current medical standards. MINOCA patients were identified if having the diagnosis of STEMI or NSTEMI with non-obstructive CAD visualized in angiography, and no previous coronary revascularization [4]. MINOCA patients’ characteristics were compared with obstructive CAD patients. Statistical analysis was based on standard descriptive statistics. Categorical variables were presented as percentages. Continuous variables were expressed as median and interquartile range (IQR). Normality was assessed by the Kolmogorov–Smirnoff–Lillifors (KLS) test. Equality of variances was assessed using the Levene’s test. Differences between groups were compared using the Student’s or the Welch’s t-test depending on the equality of variances for normally distributed variables. The Mann–Whitney U test was used for non-normally distributed continuous variables. Ordinal variables were compared using the Cochran–Armitage test. Nominal variables were compared by the Pearson’s chi-squared test or by the Fisher’s exact test if 20% of cells had expected count < 5. The level of statistical significance was set at p < 0.05. All analyses were carried out with JMP®, Version 12.2.0 (SAS Institute Inc., Cary, NC, USA).

## Results

**Fig. 1:**
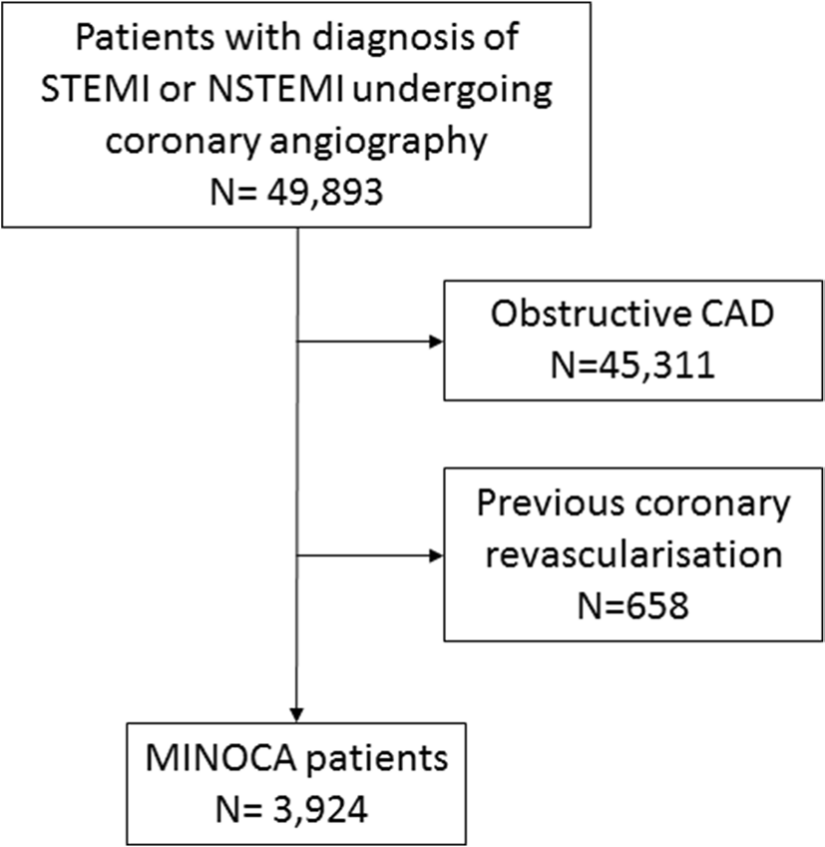
Study cohort. Patients with myocardial infarction enrolled into the ORPKI registry in 2016

In 2016, 49,893 patients with STEMI or NSTEMI entered the ORPKI registry. MINOCA was identified in 3924 (7.8%) patients (Fig. 1). Among these patients, NSTEMI was more frequent than STEMI. Time from pain onset to first medical contact and from contact to angiography was longer in the MINOCA group (median 240 vs. 180 min; 330 vs. 145 min; respectively; p < 0.0001 for both). Patients with MINOCA were younger and more often women with a lower rate of CAD risk factors comparing to patients with obstructive CAD. Heart failure symptoms (Killip class > 1) and cardiac arrest before angiography were less frequent in MINOCA patients than in the obstructive CAD group. MINOCA patients were less likely to receive antiplatelet and antithrombotic treatment before angiography. Additional physiological assessment and intravascular imaging was marginally (< 1%) used in both groups. Myocardial bridges and coronary fistulas were more frequent in the MINOCA group (Table 1). Periprocedural mortality was lower in MINOCA group (0.13% vs. 0.95%; p < 0.0001).

**Table 1:** Characteristics of studied groups

	MINOCA (n = 3924)	Obstructive CAD (n = 45,969)	p-value
Type of MI at presentation			< 0.0001
STEMI (%)	22	48.9	
NSTEMI (%)	78	51.1	
Age [years, median (25th–75th percentile)]	65.00 (55.00;75.00)	67.00 (59.00;76.00)	< 0.0001
Female (%)	52.0	32.9	< 0.0001
Diabetes mellitus (%)	13.1	22.2	< 0.0001
Smoking (%)	15.1	25.2	< 0.0001
Arterial hypertension (%)	56.4	64.3	< 0.0001
Chronic kidney disease (%)	4.4	5.9	< 0.0001
Previous stroke (%)	2.7	3.7	0.0014
Previous MI (%)	4.4	19.2	< 0.0001
COPD (%)	3.0	2.9	0.7
Killip class on admission			< 0.0001
I (%)	93.8	86.2	
II (%)	4.0	8.3	
III (%)	1.2	2.7	
IV (%)	1.0	2.8	
Cardiac arrest before angiography (%)	0.13	0.54	< 0.001
ASA before angiography (%)	53.7	63.4	< 0.0001
Unfractionated heparin before angiography (%)	30.3	39.2	< 0.0001
P2Y_12_ inhibitor before angiography			< 0.0001
Clopidogrel (%)	36.8	46.2	
Ticagrelor (%)	1.1	1.8	
Prasugrel (%)	0.03	0.2	
Additional coronary artery assessment			
FFR (%)	0.38	0.08	< 0.0001
IVUS (%)	0.11	0.13	0.8
OCT (%)	0.08	0.02	0.06
Myocardial bridge (%)	2.2	0.36	< 0.0001
Coronary fistulas (%)	0.18	0.03	< 0.001

*STEMI* ST-segment elevation myocardial infarction, *NSTEMI* non-ST-segment elevation myocardial infarction, *MI* myocardial infarction, *COPD* chronic obstructive pulmonary disease, *ASA* acetylsalicylic acid, *FFR* fractional flow reserve, *IVUS* intravascular ultrasound, *OCT* optical coherence tomography

## Discussion

In the current analysis of the large-scale national database, we showed that MINOCA patients represent a significant proportion of MI patients referred to invasive assessment in Poland. We found MINOCA in about 8% of patients which is similar to the recently published report from SWEDEHEART Registry (about 8%) as well as a meta-analysis of clinical studies (about 6%) [5, 6].

Importantly, MINOCA is just an initial and general diagnosis which does not describe underlying pathophysiology. The potential pathophysiological mechanisms of clinical scenario when MI may be diagnosed according to the definition but there is a lack of the obstructive coronary artery disease are quite complex. These include both coronary and non-coronary pathologies. The coronary causes comprise of several different mechanisms. Thromboembolism may be an underlying pathological factor by itself or may be caused by plaque rupture or coronary spasm. This includes also thrombotic disorders (hereditary or acquire) [6]. Plaque disruption may be caused by erosion, ulceration, plaque rupture, and intraplaque hemorrhage. Coronary artery spasm may be present not only due to endogenous causes but may be provoked by exogenous substances like cocaine [7, 8]. Non-coronary etiologies are also frequent in MINOCA patients. It is important to recognize well-defined diseases with described etiopathologies like myocarditis, pulmonary embolism or Takotsubo cardiomyopathy in patients initially described as MINOCA. Importantly, some of those causes are treatable, so well planned diagnostics seems to be crucial for the final diagnosis, treatment selection and outcome of these patients. According to 2017 European Society of Cardiology Clinical Practice Guidelines on STEMI, failure to identify the underlying cause may result in inadequate and inappropriate therapy in MINOCA patients [1]. The diagnostic algorithm based on suspected diagnosis and corresponding diagnostics modalities (non-invasive and invasive) was proposed. This includes myocarditis (with echocardiography, cardiac magnetic resonance and endomyocardial biopsy), coronary epicardial/microvascular etiology (with intravascular ultrasound (IVUS), ergonovine/acetylocholine test, pressure/doppler wire), myocardial disease (with echocardiography and cardiac magnetic resonance), pulmonary embolism (with D-dimer, CT scan, thrombophilia screening) and type 2 MI (with extracardiac investigation) [1]. In the individual patients’ data meta-analysis of MINOCA patients diagnosed with cardiac magnetic resonance one-third of patients had myocarditis, whereas 21% had infarction in delayed enhancement imaging [9]. Intracoronary imaging is important in selected cases since plaque rupture, ulceration, erosion or intraplaque hemorrhage are rarely visible in angiography in non-obstructive CAD. Reynolds et al. showed that plaque disruption confirmed by IVUS was observed in 38% of women with MINOCA. Interestingly, in some cases, plaque rupture was identified by IVUS even in angiographically normal-appearing segments [10]. In addition, in patients with MINOCA invasive coronary provocative tests may be considered. Montone et al. showed acetylcholine and ergonovine tests to be safe in patients with MINOCA and suspected coronary vasomotor abnormalities. Moreover, test results correlated with clinical symptoms and outcome in follow-up [11]. In the meta-analysis of MINOCA studies, coronary artery spasm was inducible in 27% of patients [6]. In our analysis (patients undergoing coronary angiography in the year 2016) additional invasive imaging during coronary angiography was marginally used. However, the current upgrade of the reimbursement program in Poland should solve this diagnostics limitation.

In line with previous reports, we found MINOCA patients to be younger, rather with an initial diagnosis of NSTEMI than STEMI and to be more often female comparing to patients with obstructive CAD. Our analysis also showed that MINOCA patients had a lower risk profile concerning other CAD risk factors. However, in meta-analysis of MINOCA studies there was no significant difference in arterial hypertension, diabetes mellitus, smoking, and family history of CAD comparing to obstructive CAD patients [6].

Patients with MINOCA have lower mortality comparing to obstructive CAD patients with MI in 12-month follow-up. However, in-hospital mortality of about 1% and 3.5% at 12-month is still high especially as compared to stable non-MI patients with normal coronaries in angiography [6]. This underlines the need for precise diagnosis and dedicated treatment of MINOCA patients. In our analysis, only periprocedural mortality data were available with significantly lower rates found in MINOCA comparing to MI patients with obstructive CAD.

Presented analysis has several limitations. Angiograms were assessed locally by operators, and not by an independent image analysis core laboratory. The ORPKI for the moment does not collect data beyond the cathlab. So, it was impossible to assess further diagnostics done during hospitalization and after discharge and to analyze data according to final diagnosis as well as provide event rates at follow-up. In addition, there is a potential bias from unmeasured confounding factors not included in this analysis. Despite all these limitations our study reflects the outcome of a “real-world”. Thus, data could be extrapolated to the general population.

## Conclusions

Patients with MINOCA represent a significant proportion of MI patients in Poland. Due to multiple potential causes, MINOCA should be considered as a working diagnosis after coronary angiography and further efforts should be taken to define the cause of MI in each patient.
